# Highly efficient acousto-optic modulation using nonsuspended thin-film lithium niobate-chalcogenide hybrid waveguides

**DOI:** 10.1038/s41377-022-00840-6

**Published:** 2022-05-20

**Authors:** Lei Wan, Zhiqiang Yang, Wenfeng Zhou, Meixun Wen, Tianhua Feng, Siqing Zeng, Dong Liu, Huan Li, Jingshun Pan, Ning Zhu, Weiping Liu, Zhaohui Li

**Affiliations:** 1 Department of Electronic Engineering, College of Information Science and Technology, Jinan University, 510632 Guangzhou, China; 2 Guangdong Provincial Key Laboratory of Optoelectronic Information Processing Chips and Systems, Sun Yat-sen University, 510275 Guangzhou, China; 3 State Key Laboratory for Modern Optical Instrumentation, College of Optical Science and Engineering, International Research Center for Advanced Photonics, Zhejiang University, Zijingang Campus, 310058 Hangzhou, China; 4 Institute of Semiconductor Science and Technology, Guangdong Engineering Technology Research Center of Low Carbon and New Energy Materials, South China Normal University, 510631 Guangzhou, China; 5 Southern Marine Science and Engineering Guangdong Laboratory (Zhuhai), 519000 Zhuhai, China

**Keywords:** Optics and photonics, Other photonics

## Abstract

A highly efficient on-chip acousto-optic modulator is as a key component and occupies an exceptional position in microwave-to-optical conversion. Homogeneous thin-film lithium niobate is preferentially employed to build the suspended configuration for the acoustic resonant cavity, with the aim of improving the modulation efficiency of the device. However, the limited cavity length and complex fabrication recipe of the suspended prototype restrain further breakthroughs in modulation efficiency and impose challenges for waveguide fabrication. In this work, based on a nonsuspended thin-film lithium niobate-chalcogenide glass hybrid Mach–Zehnder interferometer waveguide platform, we propose and demonstrate a built-in push-pull acousto-optic modulator with a half-wave-voltage-length product *V*_*π*_*L* as low as 0.03 V cm that presents a modulation efficiency comparable to that of a state-of-the-art suspended counterpart. A microwave modulation link is demonstrated using our developed built-in push-pull acousto-optic modulator, which has the advantage of low power consumption. The nontrivial acousto-optic modulation performance benefits from the superior photoelastic property of the chalcogenide membrane and the completely bidirectional participation of the antisymmetric Rayleigh surface acoustic wave mode excited by the impedance-matched interdigital transducer, overcoming the issue of low modulation efficiency induced by the incoordinate energy attenuation of acoustic waves applied to the Mach–Zehnder interferometer with two arms in traditional push-pull acousto-optic modulators.

## Introduction

The acousto-optic (AO) interaction is a multiphysics coupling process in which radiofrequency (RF)-driven acoustic waves in a medium can change the localized refractive index of the waveguide, thereby manipulating photons^1–8^; this process has stimulated ingenious applications in many areas, such as coherent quantum transduction^9^, nonreciprocal light transmission^10,11^, modulation^12^, frequency shifting^13^, signal processing^14,15^, beam deflection^16,17^, and filtering^18^. Presently, traditional AO devices based on bulk crystal materials have weak energy confinement abilities for both photons and phonons, leading to a low AO interaction strength^19^. Compared with bulk materials, photonic integrated circuits (PICs) allow surface acoustic waves (SAWs) to be well confined within the thin film used to disturb the guided light waves, exhibiting a high energy overlap within the wavelength scale. Therefore, SAWs are desirable, effective tools to achieve a very high AO modulation efficiency and can further enable a diversity of functionalities in PICs.

SAWs are generated by interdigital transducers (IDTs) placed over the thin-film piezoelectric materials in PICs. SAW-coupled high-performance AO modulation requires a low-loss optical waveguide and a high-efficiency IDT, which are simultaneously integrated into an on-chip optoelectronic platform by judiciously engineering the configurations of the optical and acoustic components and the relative position between them. With the development of fabrication technologies for thin-film piezoelectric materials, on-chip AO modulators have been accordingly demonstrated by homogeneously integrating waveguides and IDTs in the same optical membranes, such as gallium arsenide (GaAs)^20,21^, polycrystalline aluminum nitride^5^, lithium niobate-on-insulator (LNOI)^22–25^, and LN-on-sapphire^26^. In particular, as one of the most promising AO interaction platforms, thin-film lithium niobate (TFLN) provides great potential for the realization of high-performance AO modulators due to its superior advantages in piezoelectric transduction and electro-optical conversion^27–32^. However, limited by the low optomechanical coupling coefficients, weak AO modulation efficiencies have become one of the bottlenecks for microwave-to-optical conversion in 5G/6G and emerging quantum signal processing applications.

To meet the challenges arising from the AO modulation efficiency, various acoustic cavity configurations are commonly considered to enhance the amplitudes of acoustic waves. In nonsuspended TFLN, acoustic resonant cavities consisting of a pair of metal gratings are engineered to increase the SAW and AO interaction^23^. In fact, the long grating dimension of the reflectors and the diffraction effect increase the acoustic wave loss, leading to low acoustic Q-factors and large *V*_*π*_*L*. To significantly improve the AO modulation efficiency, a free-standing LN thin film is a preferred, feasible solution to form a standing wave between two open slits with a distance of tens of μm so that the excited weak acoustic wave can be greatly amplified to enhance the overlap factor between the optical and acoustic modes. However, because of the need for suspended LN acoustic resonator construction and for the placement of the sensitive optical waveguide over the suspended acoustic resonator, there are stringent requirements on the sophisticated fabrication techniques, especially for heterogeneous-integration waveguides^22,24^. Acquiring a high AO modulation efficiency without a suspended acoustic resonator has become an important topic for microwave-to-optical conversion.

Generally, the IDT of an AO modulator with a Mach–Zehnder interferometer (MZI) configuration is placed outside the two arms of a nonsuspended waveguide to modulate the refractive index of a single arm. Since the SAW excited by the IDT propagates in two opposite directions, only 50% of the effective energy within the total converted acoustic wave can ideally reach the waveguide and participate in the interaction with light waves under the single arm modulation mechanism, resulting in a quite low modulation efficiency. Although push-pull MZI modulation configurations (odd multiple of half the acoustic wavelength) have been proposed by carefully designing the distance between the two arms of the MZI and optimizing the structures of the metal grating reflectors, the diffraction effect of metal grating and the reconstructed acoustic mode introduced by the SAW reflection and the interference originating from waveguide sidewalls inevitably lead to much more SAW energy being wasted and cannot enable a modulation efficiency with a twofold enhancement, defeating the initial purpose of the double arm modulation^23,33^.

To overcome the above problems, a novel built-in push-pull AO modulation configuration based on the antisymmetric SAW mode, for which the optimized IDT is placed inside the two arms of the designed MZI waveguide, is theoretically proposed and experimentally demonstrated based on nonsuspended TFLN in our work. This configuration can suitably relax the fabrication complexities of the optical waveguide and the IDT, enabling a modulation efficiency that is twice as efficient as the single arm configuration due to the employment of 100% of the bidirectional acoustic energy in principle. In addition, considering that amorphous chalcogenide glasses (ChGs), which are soft infrared waveguide materials, have a better photoelastic coefficient that is nearly two times that of TFLN^34–37^, the TFLN-ChG hybrid waveguide platform is adopted to facilitate the improvement of the AO modulation efficiency. Most of the optical energy is designed to be confined in the ChG rectangular waveguide to take full advantage of the dominant photoelastic effect. Proper engineering of the geometry of the nonsuspended TFLN-ChG hybrid waveguide combined with a precisely defined IDT can not only significantly increase the double arm modulation efficiency in the push-pull MZI configuration but can also avoid direct etching of TFLN; thus, potential prospects in microwave-to-optical conversion are presented. In the experiments, the *V*_*π*_*L* of our AO modulator with the double arm configuration based on the nonsuspended TFLN-ChG hybrid MZI is demonstrated to be as low as 0.03 V cm, which is one order of magnitude smaller than that of the nonsuspended homogeneous TFLN counterpart^24^. To the best of our knowledge, this is the first built-in push-pull modulator implemented in on-chip AO devices in which the modulation efficiency exceeds that of AO modulators with acoustic cavities^22–24^. To further present the low power consumption feature, we characterize the optical and RF sidebands of the device and verify the efficient transmission of an on-off modulated signal. Combined with the simple fabrication processes and high-performance modulation efficiency, our built-in push-pull AO modulator is expected to show excellent characteristics in on-chip RF-driven optical isolators^38^ and integrated analog optical computing systems^39^.

## Results

### Device design

**Fig. 1:**
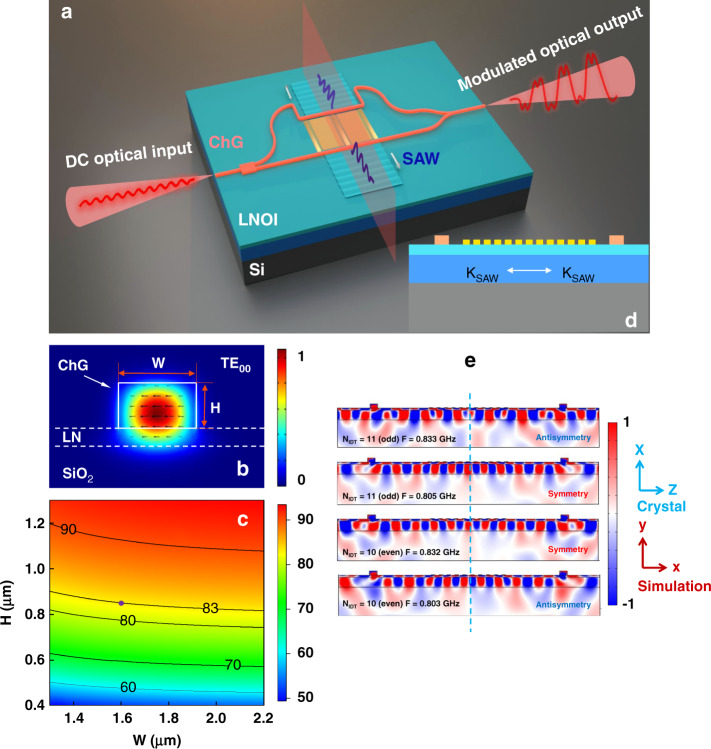
Design of a built-in push-pull AO modulator based on a nonsuspended TFLN-ChG hybrid waveguide platform. **a** Schematic diagram of the proposed device. The MZI waveguide is etched on the top of the Ge_25_Sb_10_S_65_ film (orange), which is deposited on the LNOI wafer (light blue). The IDT made of Au electrodes (yellow) is evaporated in the region between the two arms. In principle, the direct current (DC) optical input is modulated into a distorted sinusoidal time-domain signal via a varied SAW. **b** Electric field of the fundamental TE mode. W and H are the width and height of the ChG rectangular waveguide, respectively. **c** Relation between the energy confinement factor Γ in the ChG waveguide and waveguide geometry. **d** Cross-sectional view of the proposed device. The colors of the waveguide materials are the same as in (**a**). **e** Numerical simulation results of the dominant S_xx_ strain components of the SAW modes in the heterogeneous-integration waveguide platform with the double arm modulation configuration. The upper two pictures are the antisymmetric and symmetric acoustic modes corresponding to 5.5 pairs of IDT fingers (N_IDT_ = 11), and the lower two are the symmetric and antisymmetric acoustic modes corresponding to five pairs of IDT fingers (N_IDT_ = 10). The color scale bars are normalized.

A schematic diagram of the proposed device is shown in Fig. 1a. An AO modulator composed of an MZI waveguide and an IDT inside the two arms of the MZI is constructed on a nonsuspended TFLN-ChG hybrid waveguide platform. X-cut LNOI with a thickness of 400 nm is chosen to excite a bidirectional Rayleigh SAW propagating in the Z direction using the piezoelectric effect and IDT. The MZI ridge hybrid waveguide is composed of a Ge_25_Sb_10_S_65_ (one of the ChGs) rectangular waveguide and a TFLN slab because the refractive index of amorphous Ge_25_Sb_10_S_65_ (*n* = 2.23) is very close to the refractive index of the LN crystal (*n*_e_ = 2.13) at 1550 nm. The similar optical refractive indices of Ge_25_Sb_10_S_65_ and LN enable the fundamental transverse-electric (TE) mode to be simultaneously confined in the Ge_25_Sb_10_S_65_ and LN layers, as shown in Fig. 1b.

To determine the structural parameters of the built-in push-pull AO modulator, we conduct numerical simulations for the optical and acoustic modes in the nonsuspended TFLN-ChG hybrid waveguide platform. Herein, the thickness of TFLN is fixed at 400 nm. The thickness of the oxide layer is 2 μm. The geometries of the ChG rectangular waveguides are varied to engineer proper parameters to adequately employ their photoelastic property advantages. Figure 1c shows that the energy confinement factors (Γ) of the fundamental TE modes in the ChG rectangular waveguides greatly depend on H rather than W. As H increases, the mode energy gradually concentrates in the ChG material. In our experiments, an H of 850 nm and a W of 2.05 μm are set to support the AO interaction because an overlarge sidewall height would bring increased scattering loss. In the AO interaction area, a taper is added to convert the waveguide width from 2.05 to 1.6 μm to match the half wavelength of the acoustic wave (Λ = 3.2 μm) to achieve the maximum AO overlap. The Γ of the 1.6 μm-wide ChG rectangular waveguide supporting the TE_00_ mode is calculated to be 83%, which is highlighted by the purple dot in Fig. 1c. An optimized multimode interference (MMI) coupler and Y-combiner are placed at the input and output ports of the hybrid MZI to uniformly route the photon energy while decreasing the insertion loss of the device. Four ultracompact 90° ChG bending waveguides with an effective radius of 10 μm are inversely designed based on freeform curves^40^. The loss of each 90° bending waveguide is experimentally measured to be approximately 0.25 dB. The introduction of an ultracompact 90° bending waveguide can facilitate precise control of the distance between the MZI arms and the flexibly designed IDT in the interaction area, avoiding the acoustic wave dissipation induced by a long propagation distance.

Figure 1d shows a cross-sectional image of the TFLN-ChG hybrid MZI-based AO modulator in the interaction area, denoted by the light red plane in Fig. 1a. To reveal the acoustic mode excited by the built-in IDT in the two-arm waveguides, we simulate the dominant S_xx_ strain components at an acoustic frequency (F) of nearly 0.833 GHz and 0.805 GHz, corresponding to 5.5 (finger number N_IDT_ = 11, odd) and 5 (N_IDT_ = 10, even) pairs of IDT fingers, respectively, as presented in Fig. 1e. Herein, in contrast to the experimental IDT, a reduced finger number is set in the eigenmode simulation to reasonably reflect the acoustic mode distribution because the finger number difference does not influence the sign relation of the acoustic mode in the two arms of the MZI. The IDT with an odd number of fingers yields an antisymmetric strain field in the two-arm waveguides at 0.833 GHz, but at the low frequency of 0.805 GHz, the IDT with the same configuration exhibits a symmetric acoustic mode. In contrast, the IDT with an even number of fingers generates a symmetric strain field at 0.832 GHz, and the same IDT exhibits an antisymmetric acoustic mode at 0.803 GHz. This means that the signs of the strain fields in the two-arm waveguides strongly depend on the number of fingers of the IDT at the specific acoustic frequency. The simulation results of the S_ZZ_ strain component have similar variations (see Supplementary note 1). By reasonably engineering the IDT structure with an odd number of fingers, opposite refractive index changes can be realized in the two-arm waveguides, satisfying the requirement of the push-pull AO modulator in principle. To maximize the microwave-to-acoustic conversion, the structural parameters of the IDT must be engineered to satisfy the impedance matching at a fixed LN film interface. According to previous tests, the IDT with ~50 pairs of fingers is helpful in achieving deep sharp reflection at ~0.844 GHz, presenting high-Q acoustic resonance and high-efficiency energy conversion. In addition, considering the requirement of the push-pull AO modulator, an IDT with an odd number of fingers is required to yield an antisymmetric strain field in the two-arm waveguides. Therefore, combined with the perspective of impedance matching, we design an IDT with 50.5 pairs of fingers (N_IDT_ = 101) to efficiently excite the SAW in the following experiments. To reveal the reasonability of the designed TFLN-ChG hybrid MZI AO modulator, the numerical simulation results of AO interactions are presented in Supplementary note 2. Herein, the photoelastic coefficients of amorphous Ge_25_Sb_10_S_65_ film are set to be *p*_11_ ≈ *p*_12_ ≈ 0.238. The detailed calculation processes are shown in Supplementary note 5.

### Device characterization

**Fig. 2:**
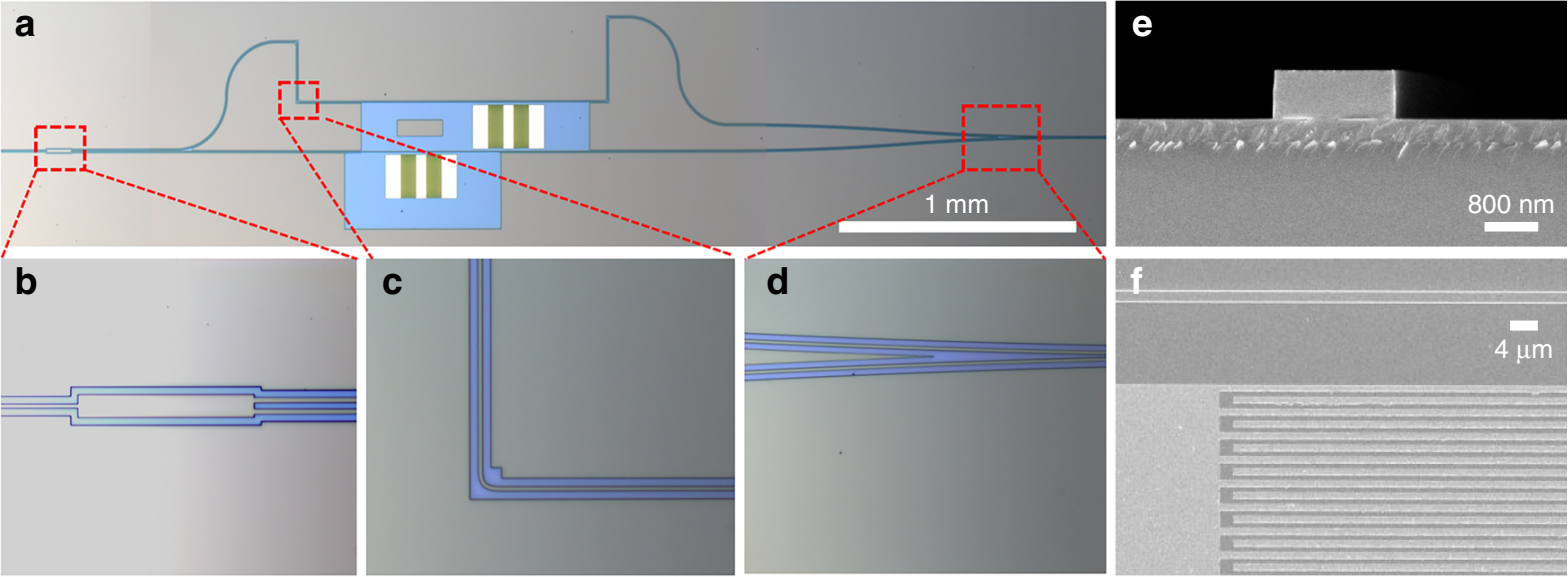
Configuration of the built-in push-pull AO modulator over the nonsuspended TFLN-ChG hybrid waveguide platform. **a** Microscope image of a TFLN-ChG hybrid MZI-based AO modulator. The MZI is designed to allow careful switching between the single arm and double arm modulation mechanisms. **b** Zoomed-in microscope images of the 1 × 2 MMI coupler, (**c**) 90° bending waveguide, and (**d**) Y-combiner. **e** Cross-sectional SEM image of the modulated waveguide. **f** SEM image of the modulation area including the IDT and waveguide.

Figure 2a shows optical microscopy images of the fabricated device A (see Supplementary note 4). For reference, two identical IDTs are placed inside and outside the two-arm waveguides, forming the double arm and single arm modulation configurations, respectively. To ensure that the SAW excited by the outside IDT only interacts with the lower waveguide, we design an unetched ChG patch along the propagation path of the SAW. Due to the strong absorption of the ChG material for the SAW, the acoustic wave cannot reach the upper arm of the MZI. The three electrodes of the built-in IDT are ground-signal-ground. Two completely axisymmetric IDT components along the middle signal electrode with 50.5 pairs of fingers constitute the built-in IDT. The generated SAWs on the two IDT components have the same symmetry. The widths of the ground and signal electrodes are set to 60 μm and 40 μm, respectively, and the aperture width is 2 × 60 μm. The width of each IDT finger is 0.8 μm. Accordingly, the period of the IDT is calculated to be 4 × 0.8 μm. This value is chosen to match the designed ChG waveguide with a width of 2.05 μm because an acoustic wave with a half wavelength approaching the width of the ChG waveguide is beneficial to acquire a strong AO interaction^12^. Figure 2b, c, and d are magnified microscope images of the 1 × 2 MMI coupler, ultracompact 90° bending waveguide, and Y-combiner, respectively. The length and width of the MMI coupler are designed to be 97 and 11.5 μm, respectively. Figure 2e and f are scanning electron microscopy (SEM) images of the modulated waveguide showing a cross-sectional view and the IDT details. The width of the ChG waveguide is measured to be 1.6 μm, and the actual sidewall angle is estimated to be 89.6°. The gaps between the IDTs and two-arm waveguides are designed to be 11.8 μm. To adjust the gap, four ultracompact 90° ChG bending waveguides with an effective radius of 10 μm are chosen to build an unbalanced MZI configuration while maintaining the appropriate insertion loss and small footprint. As a more common design, the S-bend waveguide with small bending radius can substitute the 90° bending waveguide to further decrease the insertion loss of the MZI AO modulator.

### Microwave-to-acoustic conversion

**Fig. 3:**
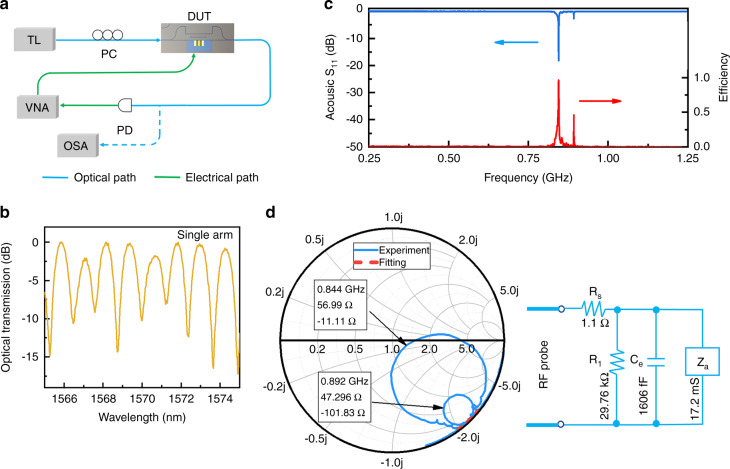
Characterization of microwave-to-acoustic conversion in the single arm-modulated AO modulator. **a** Schematic diagram of the device measurement system. **b** Optical transmission spectrum of an MZI-based AO modulator with the single arm modulation configuration. **c** S_11_ reflection spectrum and conversion efficiency of the fabricated IDT. **d** Smith chart of the fabricated IDT and the corresponding effective circuit model. TL: tunable laser, VNA: vector network analyzer, DUT: device under test, PC: polarization controller, PD: photodiode, OSA: optical spectrum analyzer.

Figure 3a shows a schematic diagram of the experimental setup of the on-chip AO modulator. We experimentally characterize the MZI-based AO modulator using a tunable C-band laser, a vector network analyzer, and a photoreceiver with a sensitivity of 800 V/W. A fiber polarization controller is used to adjust the fundamental TE mode fed into the waveguide, and a pair of lens fibers with a mode field diameter of 3 μm are utilized for the edge-coupling between the input and output light and device. Herein, given that device A has inadequate impedance matching due to the process deviation, we mainly discuss the modulation characteristics of device B and device C, corresponding to the single-arm and double-arm modulator configurations, respectively. The fiber-to-fiber insertion loss of the AO modulator is measured to be ~16 dB, and the edge-coupling loss is 5.5 dB/facet, illustrating an on-chip loss of 5 dB, which mainly benefits from the low sidewall roughness and high-quality ChG membrane deposition. Herein, the further reduction of the insertion loss of the device can be attributed to the engineering of the inverse taper configuration in the end faces of the hybrid waveguide. The normalized optical transmission spectrum of device B presents periodic interference with a free spectral range of nearly 1.2 nm, as shown in Fig. 3b, which is consistent with the natural optical path difference of the designed MZI. As depicted in the transmission spectrum, the extinction ratio (ER) of the device is ~17 dB at ~1573 nm.

We evaluate the microwave-to-acoustic conversion by measuring the microwave reflection coefficient (S_11_) spectrum of the fabricated IDT, which exhibits two resonance dips within the 0.25−1.25 GHz range, as shown in Fig. 3c. The deep reflection valley is located at 0.844 GHz, and 1-|S_11_|^2^ is calculated to be 98.5%, which represents the ratio of the RF power loaded on the IDT to the input RF power. The Q-factor of the acoustic resonance is estimated to be 265, corresponding to a linewidth of 3.19 MHz. Although the Q-factor of the acoustic resonance is low, a significantly increased RF power loaded on the IDT facilitates the effective generation of SAW. The experimentally observed dominant acoustic wave at 0.844 GHz belongs to the antisymmetric Rayleigh SAW, which agrees well with the acoustic mode displayed in the top panel of Fig. 1e. The slight variation in frequency may be caused by the fabrication deviation and finger number difference of the IDT in the two cases. In the experiments, we can see acoustic waves excited with frequency up to 2.4 GHz. The Smith chart shows that the designed IDT greatly satisfies the impedance matching condition at 0.844 GHz, as emphasized by the large circle in Fig. 3d. Accordingly, the complex impedance of the IDT at 0.844 GHz is measured to be 56.99-*j*11.11 Ω, approaching the standard 50 Ω. To precisely estimate the microwave-to-acoustic conversion efficiency, a Butterworth-Van Dyke model is introduced to reveal the energy consumption in load Z_a_, which is the available acoustic energy in the DA modulation configuration, by fitting the nonresonant normalized complex impedances in the Smith chart^41^, as denoted by the red dotted line in Fig. 3d. Herein, R_s_ accounts for the contact resistance between the RF probe and IDT fingers, and R_1_ and C_e_ account for the effective leakage resistance and capacitance in the IDT region, respectively. The detailed calculation processes are described in Supplementary note 3. By estimating the ratio of the electrical power consumed in load Z_a_ to the RF power loaded on the IDT at the different microwave frequencies, we can acquire the microwave-to-acoustic conversion efficiency of the designed IDT, as shown by the red line in Fig. 3c. As a result, the peak conversion efficiency is calculated to be 0.96 at 0.844 GHz. The electromechanical coupling efficiency K^2^ is estimated to be 0.692%. Although the XZ direction may not be the most suitable crystal orientation due to the anisotropic feature of TFLN, reasonably engineering the impedance matching of IDT enables us to realize a 96% conversion efficiency in the microwave-to-acoustic conversion. Compared with the suspended TFLN proposed in Refs. ^22,24^, the flexible fabrication of the proposed IDT over the nonsuspended TFLN ensures highly efficient electromechanical conversion in our experiments.

### AO modulation comparison

**Fig. 4:**
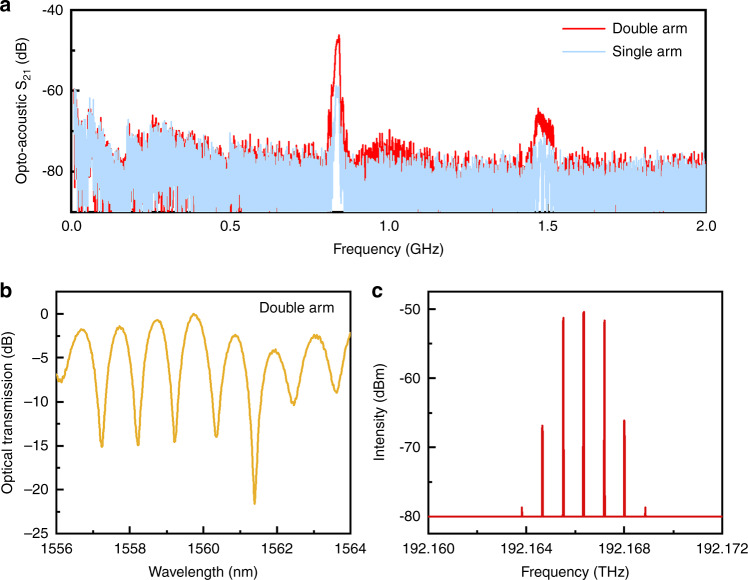
AO modulation. **a** S_21_ spectra of the TFLN-ChG hybrid MZI-based AO modulators with single-arm and double-arm modulation configurations. **b** Normalized optical transmission spectrum of the AO modulator with the double arm configuration. **c** Measured optical sidebands in the push-pull AO modulator at an RF power of 15 dBm.

We characterize the AO modulation of the device by the opto-acoustic S_21_ spectrum, where driving Port 1 of the VNA is connected to the IDT and detecting Port 2 is connected to the photoreceiver (see Fig. 3a). The S_21_ spectrum shown in Fig. 4a has two significant peaks in the frequency range of 0−2 GHz, indicating that the microwave-to-optical conversion is enhanced at these frequencies. The strong opto-acoustic response is positioned at 0.844 GHz, corresponding to the sharp dip displayed in the S_11_ spectrum (Fig. 3c). Compared with device B with the single arm modulation configuration, the built-in push-pull AO modulator (device C) obtains a nearly 12 dB improvement in S_21_ under an RF power of 0 dBm, greatly demonstrating the effectiveness of double arm modulation. The enhanced S_21_ benefits from the increase in the ER at ~1561 nm in the transmission spectrum of device C, as shown in Fig. 4b. In other words, 12 dB enhancement in S_21_ is mainly attributed to 3.5 times slope variation at DC bias points in terms of both cases.

To further quantify the AO modulation characteristics, we extract the *V*_*π*_ of the proposed nonsuspended MZI-based AO modulator from the experimental measurement of the S_21_ spectrum by the following equation [Eq. 1]:^24^

1 $$V_\pi {{{\mathrm{ = }}}}\frac{{\pi R_{PD}I_{rec}}}{{\left| {S_{21}} \right|}}$$

where *R*_*PD*_ is the sensitivity of the photoreceiver and *I*_*rec*_ is the DC optical power at the bias point with a π/2 phase difference between the two arms of an MZI. Herein, the bias points of devices B and C are at ~1573 nm and 1561 nm, respectively. By choosing bias point in the transmission spectrum, *I*_*rec*_ is measured to be −23 dBm (−24 dBm) for device C (device B). From the S_21_ spectrum, device C (device B) has an S_21_ of −46 dB (−58.8 dB) at 0.844 GHz. The *V*_*π*_ of device C (device B) is thus calculated to be 2.5 V (8.68 V), corresponding to *V*_*π*_*L* = 0.03 V cm (0.1 V cm) due to the modulation length of 120 μm, which shows that the built-in push-pull AO modulator has a threefold enhancement in the modulation efficiency compared with the single arm modulator. To the best of our knowledge, this is the state-of-the-art on-chip AO modulator based on nonsuspended TFLN. The excellent modulation efficiency is attributed to the highly efficient employment of a bidirectionally propagating SAW combined with the outstanding photoelastic property of the ChG material. Considering the scattering of the acoustic wave from the ChG waveguide, precisely engineering the geometry of the ChG waveguide is beneficial to further improve the AO interaction. Detailed comparisons of the modulation characteristics are presented in Table 1. To strictly demonstrate the contribution of the proposed double arm modulation configuration, single arm- and double arm-modulated configurations are simultaneously integrated with a TFLN-ChG hybrid MZI to conduct measurements with the same optical transmission spectrum and bias point (A device), as shown in Fig. 2a. The results definitely confirm the relation of a twofold enhancement in the modulation efficiency (detailed modulation results are presented in Supplementary note 4), which is attributed to the ideally antisymmetric acoustic mode distribution in the two-arm waveguides excited by the built-in IDT.

**Table 1:** Comparison of modulation metrics for TFLN MZI-based AO modulators.

Ref./ device	Platform	Acoustic cavity	Frequency (GHz)	1-|S_11_|^2^ (%)	*L* (μm)	*α*_*p*_ (rad/√mW)	*V*_*π*_*L* (V cm)
[23]	LN^a^	√	0.11	42	1200	0.073	2.5
[33]	As_2_S_3_/SiO_2_/LN^a^	√	0.11	95	2400	0.26	0.94
[22]	LN^b^	√	3.33	64	100	0.27	0.046
[24]	LN^b^	√	1.16	19.3	45	0.54	0.019
[24]	LN	x	1.9	50	45	0.017	0.38
[24]	LN	x	1.9	90	45	0.018	0.27
Our work	SA: Ge_25_Sb_10_S_65_/LN	x	0.84	98.5	120	0.12	0.1
	DA: Ge_25_Sb_10_S_65_/LN	x	0.84	98.5	120	0.4	0.03

^a^In-plane metal grating reflectors were fabricated to construct an acoustic cavity.

^b^Suspended TFLN was etched as an acoustic cavity. The MZI modulator configurations in Refs. ^23,33^ are push-pull for Rayleigh SAWs, whereas for Refs. ^22,24^, they are single arm modulations for Lamb waves.

To further reveal the modulation characteristics of the device, we change the experimental setup to observe optical sidebands, as shown in Fig. 3a. Herein, the output light is directly injected into the high-precision spectrometer, and the frequency of the RF signal is set to 0.844 GHz. When the RF power is 15 dBm, a weak third-order sideband appears in the spectrum (Fig. 4c) using the push-pull AO modulator. The symmetric sideband spectrum closely depends on the choice of the bias point. More importantly, with the gradual increase in the RF power, we could expect to observe many more optical sidebands. As described in Ref. ^39^, the periodic sidebands can be regarded as frequency lattices, which may have potential in integrated analog optical computing applications. Meanwhile, the ER of the MZI is accordingly decreased (see Supplementary note 6), which may be caused by the enhanced acoustic wave modulation-induced energy dissipation in the MZI.

### Demonstration of an on-off modulation link

**Fig. 5:**
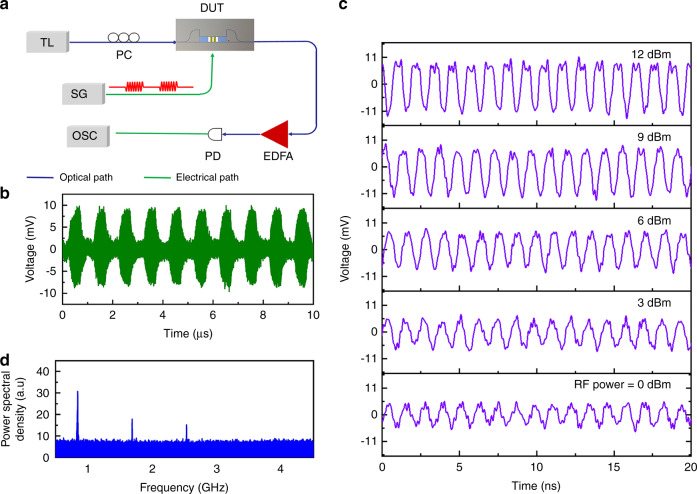
Experimental demonstration of an on-off modulation link using our built-in push-pull AO modulator. **a** Schematic diagram of an on-off modulation link. The 1 MHz on-off modulated RF signal at 0.84 GHz is delivered from a signal generator to the device under testing via a microwave probe. **b** Time-domain oscillogram of the amplitude-modulated optical wave sampled within 10 μs under 6 dBm RF power. **c** Measured time-resolved oscillograms recorded for different RF powers applied in our AO modulator. **d** Fourier transformation of the time-domain oscillogram corresponding to the input of 6 dBm RF power. SG: signal generator, EDAF: erbium-doped fiber amplifier, OSC: oscilloscope.

To demonstrate the low power consumption of the device, we construct an on-off modulation link using our nonsuspended built-in push-pull AO modulator. As shown in Fig. 5a, a microwave signal generator is used instead of the VNA to generate a square wave (on-off modulated signal) with a carrier wave frequency of 0.84 GHz and a modulation frequency of 1 MHz. The output light is amplified by an erbium-doped fiber amplifier; then, the converted electrical signal is connected to a high-speed oscilloscope. Figure 5b shows the time-domain oscillogram of the modulated optical signal from the output port of our AO modulator using only an RF power of 6 dBm. The on-off modulated RF signal is loaded onto the DC optical carrier through our push-pull AO modulator, clearly demonstrating the microwave signal transmission capability of the developed on-chip AO modulator. Time-resolved oscillograms recorded for different RF powers are shown in Fig. 5c, and gradual distortion of the sinusoidal modulated optical signal is observed with increasing RF power due to the generation of high-order sidebands. Performing Fourier transformation on the time-domain signal under an RF power of 6 dBm, as shown in Fig. 5b, we can capture an up to third-order microwave beat-note signal, as shown in Fig. 5d, demonstrating the high efficiency advantage of our developed built-in push-pull AO modulator.

## Discussion

Table 1 compares our single arm-modulated and double arm-modulated on-chip AO modulators based on nonsuspended TFLN-ChG hybrid waveguides with the present mainstream TFLN AO modulators. Good homogeneous TFLN AO modulators seriously depend on the construction of the suspended acoustic resonant cavites^22,24^, whereas our AO modulators avoid the fabrication of similar configurations, greatly relaxing the fabrication design requirements of devices. Benefiting from the excellent acoustic wave response characteristics of the TFLN-ChG hybrid waveguide platform, the prominent *V*_*π*_*L* of 0.03 V cm is experimentally demonstrated based on our proposed built-in push-pull AO modulator. As a significant figure-of-merit (FOM), the *V*_*π*_*L* of our device with the double arm configuration is one order of magnitude smaller than that of its counterpart based on a nonsuspended homogeneous TFLN waveguide platform. This impressive modulation performance substantially originates from the increased overlap integral between the optical and acoustic waves. More specifically, the highly efficient bidirectional participation of the excited antisymmetric acoustic wave in the two arms of the MZI effectively enhances the energy proportion of the Rayleigh SAW at 0.84 GHz applied in the soft ChG rectangular waveguides. As another FOM, the phase shift per unit square root microwave power *α*_*p*_ is estimated to be 0.4 rad/√mW (see Supplementary note 7), which is desirable for high-performance AO modulators. In the future, we can further improve the microwave-to-optical conversion based on the nonsuspended TFLN-ChG hybrid waveguide platform by combining an optical resonator, an optomechanical resonant cavity, and a nanobeam waveguide with built-in push-pull IDTs. Meanwhile, the effect of the acoustic wave orientation in TFLN on AO modulation will be considered to improve the performance of the device. To expand the acoustic wave frequency to several GHz, engineering of the narrow-width IDT electrode configuration is the preferred strategy. From the point of view of the optical mode, a reasonably designed ChG waveguide needs to be considered to enhance the AO interaction in the high-frequency mechanism. The modulation process driven by W-scale microwave power will be demonstrated to reveal the microwave power handling capability of the device.

In conclusion, we propose and demonstrate a built-in push-pull AO modulator based on the nonsuspended TFLN-ChG hybrid waveguide platform. Benefiting from the excellent photoelastic property of ChG and the antisymmetric acoustic mode excited by an impedance-matched IDT, the *V*_*π*_*L* of the push-pull AO modulator is measured to be as low as 0.03 V cm, reflecting the highly efficient modulation performance of our device with the double arm configuration. Compared with the AO modulator with the single arm modulation configuration, the double arm-modulated counterpart has a twofold enhancement in the modulation efficiency. In addition, the demonstration of SAW-driven AO modulation in the nonsuspended TFLN-ChG heterogeneous-integration waveguide platform simplifies the fabrication design of the device and provides sufficient degrees of freedom to flexibly design high-performance on-chip AO interaction devices. To verify the low power consumption, up to a third-order RF sideband was experimentally demonstrated via an efficient on-off modulation link. We anticipate that the development of a highly efficient on-chip AO modulator as a key component will offer opportunities for emerging RF-driven on-chip optical isolators and integrated analog optical computing devices.

## Materials and methods

### Device fabrication

The devices were fabricated on an X-cut thin-film LNOI wafer purchased from NANOLN, where the nominal thickness of the LN layer was 400 nm. We first deposited an 850 nm-thick Ge_25_Sb_10_S_65_ membrane on the LNOI wafer by the thermal evaporation method. Then, we performed electron-beam lithography (EBL) to design the MZI waveguide structure as a mask using an electron-beam resist (ARP 6200.13) and transferred the photonic waveguide onto the Ge_25_Sb_10_S_65_ film using reactive ion etching. Finally, the IDTs were fabricated through a lift-off process involving second-step EBL and gold deposition, where the thickness of the gold electrodes was 100 nm, with a 10 nm Ti adhesive layer that was previously deposited. The thickness of ARP6200.13 was controlled to 400 nm during spin coating using a spinning speed of 4000 rpm. A schematic of the fabrication processes of the device is shown in Supplementary note 8.

### Measurement methods

A C-band tunable laser (Keysight 8164B) was employed to measure the optical transmission spectrum of the MZI waveguide using a pair of lens fibers with a 3 μm mode field diameter. The characterization of the S_11_ spectra for the IDTs was conducted using a VNA (Keysight, N5225A) with the aid of a microwave probe (GGB, 40A-GSG-100-DP). Before the measurement of the S_11_ spectrum was performed, the VNA was calibrated to reset the impedance of the cable and probe. By choosing a proper bias wavelength in the transmission spectrum of the MZI, the S_21_ spectrum could be obtained by scanning the microwave frequency in the VNA when the modulated optical wave was converted into an electrical signal via a high-speed photodiode (Newport, 1544-B). The spectrum of the modulation sidebands was recorded by connecting the output fiber to a high-precision OSA (APEX, AP2088A). The acquisition of the time-domain oscillograms corresponding to the modulated optical waves was completed by delivering amplified and filtered optical signals to an OSC (LeCroy, 80 GSa/s) after photoelectrical conversion through a photodiode.

## Supplementary information

Supplementary Information

## References

[1] Balram KC, Davanco MI, Song JD, Srinivasan K (2016). Coherent coupling between radio frequency, optical, and acoustic waves in piezo-optomechanical circuits. Nat. Photon..

[2] Munk D (2019). Surface acoustic wave photonic devices in silicon on insulator. Nat. Commun..

[3] Jiang W (2020). Efficient bidirectional piezo-optomechanical transduction between microwave and optical frequency. Nat. Commun..

[4] Balram KC, Davanco MI, Song JD, Srinivasan K (2016). Coherent coupling between radiofrequency, optical and acoustic waves in piezo-optomechanical circuits. Nat. Photon..

[5] Shao L (2020). Non-reciprocal transmission of microwave acoustic waves in nonlinear parity-time symmetrical resonators. Nat. Electron..

[6] Sarabalis CJ (2021). Acousto-optic modulation of a wavelength-scale waveguide. Optica.

[7] Li H, Tadesse SA, Liu Q, Li M (2015). Nanophotonic cavity optomechanics with propagating acoustic waves at frequencies up to 12 GHz. Optica.

[8] Safavi-Naeini AH, Thourhout DV, Baets R, Laer RV (2019). Controlling phonons and photons at the wavelength scale: integrated photonics meets integrated phononics. Optica.

[9] Schuetz MJA (2015). Universal quantum transducers based on surface acoustic waves. Phys. Rev. X..

[10] Kittlaus EA, Otterstrom NT, Kharel P, Gertler S, Rakich PT (2018). Non-reciprocal interband Brillouin modulation. Nat. Photon..

[11] Kittlaus EA (2021). Electrically driven acousto-optics and broadband non-reciprocity in silicon photonics. Nat. Photon..

[12] Tadesse SA, Li M (2014). Sub-optical wavelength acoustic wave modulation of integrated photonic resonators at microwave frequencies. Nat. Commun..

[13] Fan L (2016). Integrated optomechanical single-photon frequency shifter. Nat. Photon..

[14] Dong CH (2015). Brillouin-scattering-induced transparency and non-reciprocal light storage. Nat. Commun..

[15] Kim J, Kuzyk MC, Han K, Wang H, Bahl G (2015). Non-reciprocal Brillouin scattering induced transparency. Nat. Phys..

[16] Kowel A (1981). Acousto-optics-a review of fundamentals. Proc..

[17] Shao L (2020). Integrated microwave acousto-optic frequency shifter on thin-film lithium niobate. Opt. Express.

[18] Tsai, C. S. *Guided-wave acousto-optics: interactions, devices, and applications*. (Springer, 1990).

[19] Savage N (2010). Acousto-optic devices. Nat. Photon..

[20] Fu W (2019). Phononic integrated circuit and spin-orbit interaction of phonons. Nat. Commun..

[21] de Lima MM, Beck M, Hey R, Santos PV (2006). Compact Mach-Zehnder acousto-optic modulator. Appl. Phys. Lett..

[22] Shao L (2019). Microwave-to-optical conversion using lithium niobate thin-film acoustic resonators. Optica.

[23] Cai L (2019). Acousto-optical modulation of thin film lithium niobate waveguide devices. Photon. Res..

[24] Hassanien AE (2021). Efficient and wideband acousto-optic modulation on thin-film lithium niobate for microwave-to-photonic conversion. Photon. Res..

[25] Zhu D (2021). Integrated photonics on thin-film lithium niobate. Adv. Opt. Photon..

[26] Sarabalis CJ (2020). Acousto-optic modulation in lithium niobate on sapphire. APL Photon..

[27] Qi Y, Li Y (2020). Integrated lithium niobate photonics. Nanophotonics.

[28] Boes A, Corcoran B, Chang L, Bowers J, Mitchell A (2018). Status and potential of lithium niobate on insulator (LNOI) for photonic integrated circuits. Laser Photon. Rev..

[29] Ahmed ANR, Shi S, Zablocki M, Yao P, Prather DW (2019). Tunable hybrid silicon nitride and thin-film lithium niobate electro-optic microresonator. Opt. Lett..

[30] Rao A (2016). High-performance and linear thin-film lithium niobate Mach-Zehnder modulators on silicon up to 50 GHz. Opt. Lett..

[31] Yu Z, Sun X (2020). Acousto-optic modulation of photonic bound state in the continuum. Light. Sci. Appl..

[32] Yu Z, Sun X (2021). Gigahertz Acousto-Optic Modulation and Frequency Shifting on Etchless Lithium Niobate Integrated Platform. ACS Photon..

[33] Khan MSI (2020). Extraction of elastooptic coefficient of thin-Film arsenic trisulfide using a Mach–Zehnder acousto-optic modulator on lithium niobate. J. Lightwave Technol..

[34] Zhang B (2021). On-chip chalcogenide microresonators with low-threshold parametric oscillation. Photon. Res..

[35] Lin H (2017). Chalcogenide glass-on-graphene photonics. Nat. Photon..

[36] Eggleton BJ, Luther-Davies B, Richardson K (2011). Chalcogenide photonics. Nat. Photon..

[37] Song J (2020). Ultrasound measurement using on-chip optical micro-resonators and digital optical frequency comb. J. Lightwave Technol..

[38] Sohn DB, Orsel OE, Bahl G (2021). Electrically driven optical isolation through phonon-mediated photonic Autler-Townes splitting. Nat. Photon..

[39] Zhao H., Li B., Li H. & Li M. Scaling optical computing in synthetic frequency dimension using integrated cavity acousto-optics. arxiv.org/abs/2106.08494 10.1038/s41467-022-33132-z PMC9477821 36109528

[40] Sun S (2021). Inverse Design of Ultra‐Compact Multimode Waveguide Bends Based on the Free‐Form Curves. Laser Photon. Rev..

[41] Liu Q, Li H, Li M (2019). Electromechanical Brillouin scattering in integrated optomechanical waveguides. Optica.

