# Supplementary material for: Highly efficient acousto-optic modulation using nonsuspended thin-film lithium niobate-chalcogenide hybrid waveguides
Source: Light Sci Appl. 2022 May 20;11:145. doi: 10.1038/s41377-022-00840-6 (PMC9122937; doi:10.1038/s41377-022-00840-6)
Supplement: Supplementary file 1 — Supplementary Information [file 41377_2022_840_MOESM1_ESM.docx]

Supplementary Information for Highly efficient acousto-optic modulation using nonsuspended thin-film lithium niobate-chalcogenide hybrid waveguides

Lei Wan^1,#,*^, Zhiqiang Yang^2,#^, Wenfeng Zhou^1,#^, Meixun Wen^1^, Tianhua Feng^1^, Siqing Zeng^2^, Dong Liu^2^, Huan Li^3^, Jingshun Pan^2^, Ning Zhu^4^, Weiping Liu^1^ & Zhaohui Li^2,5,*^

*^1^Department of Electronic Engineering, College of Information Science and Technology, Jinan University, Guangzhou 510632, China*

*^2^Guangdong Provincial Key Laboratory of Optoelectronic Information Processing Chips and Systems, Sun Yat-sen University, Guangzhou 510275, China*

*^3^State Key Laboratory for Modern Optical Instrumentation, College of Optical Science and Engineering, International Research Center for Advanced Photonics, Zhejiang University, Zijingang Campus, Hangzhou 310058, China*

*^4^Institute of Semiconductor Science and Technology, Guangdong Engineering Technology Research Center of Low Carbon and New Energy Materials, South China Normal University, Guangzhou 510631, China*

*^5^Southern Marine Science and Engineering Guangdong Laboratory (Zhuhai), Zhuhai 519000, China*

**Supplementary note 1. Simulation results of the S_zz_ strain component excited by the built-in IDT in the nonsuspended TFLN-ChG hybrid waveguide platform**

To understand the distribution of the Rayleigh SAW, we simulate the normalized total displacement field of the acoustic eigenmode at 0.88 GHz based on a pair of fingers due to the periodicity of the IDT electrode using the finite element method (FEM), as shown in Fig. S1(a). The normalized strain field S_xx_ and S_zz_ components can be calculated via the total displacement field. As Fig. S1(b) shows, the amplitude of the dominant S_xx_ component is twice the amplitude of S_zz_, and the S_xx_ and S_zz_ components are out of phase. To clearly reveal the phase relation of the Rayleigh SAW excited by the built-in IDT in the two-arm waveguides, the strain fields of the dominant S_xx_ component are presented in Fig. 1(e), and the results of the S_zz_ component are shown in Fig. S1(c). We find that the acoustic strain field of the S_zz_ component in the waveguide is out of phase with the S_xx_ component at the same acoustic frequency, which is consistent with the analysis of the acoustic eigenmode of the IDT. In addition, the IDT with an odd number of fingers generates an antisymmetric strain field distribution in the two-arm waveguides at 0.833 GHz, but at the low frequency of 0.805 GHz, the IDT with the same configuration exhibits a symmetric acoustic mode distribution. In contrast, the IDT with an even number of fingers generates a symmetric strain field distribution at 0.832 GHz, and the same IDT exhibits an antisymmetric acoustic mode distribution at 0.803 GHz. Similar relative phase changes in the MZI two-arm waveguides are demonstrated under the specific IDT configuration and acoustic frequency for the S_xx_ and S_zz_ strain components.


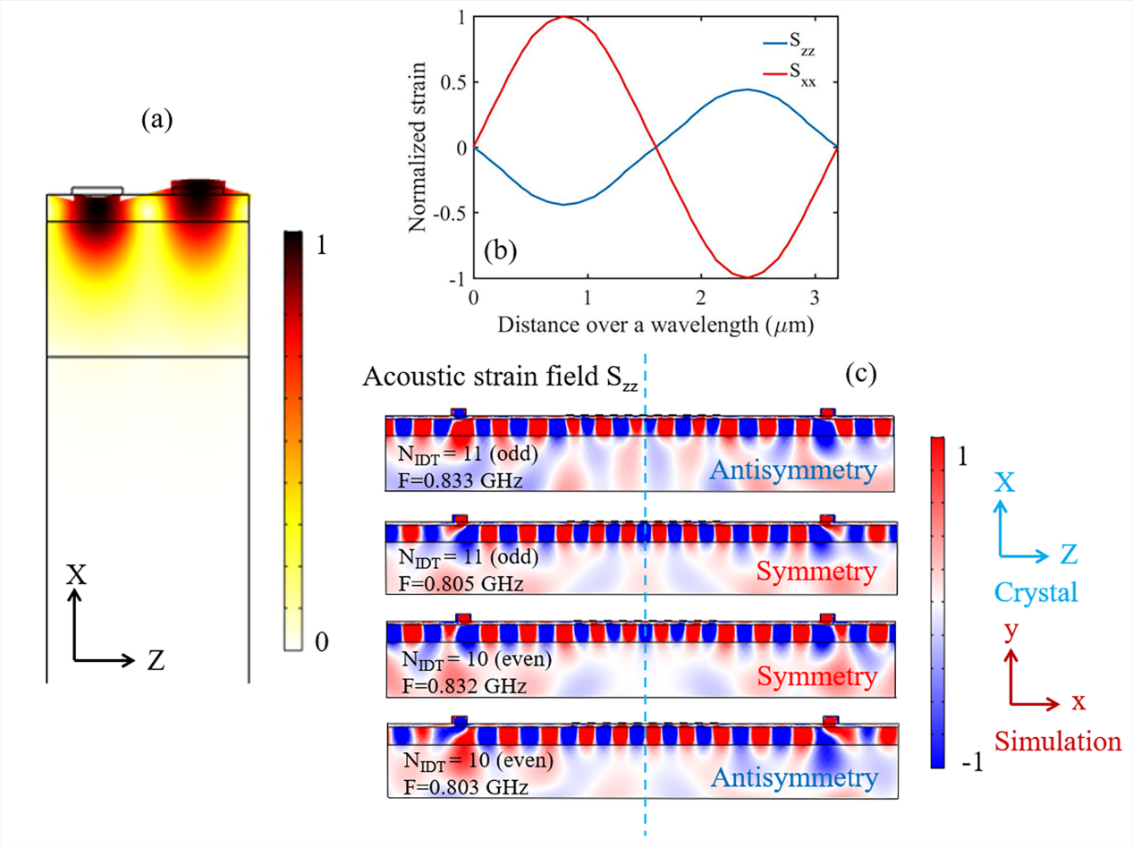


Fig. S1. **a** Normalized strain field distribution of the Rayleigh SAW eigenmode corresponding to an IDT period. **b** S_xx_ and S_zz_ components of the normalized strain field over an acoustic wave wavelength. **c** Distributions of the acoustic strain field S_zz_ in the nonsuspended TFLN-ChG hybrid two-arm waveguide platform corresponding to 5.5 (N_IDT_ = 11, odd) and 5 (N_IDT_ = 10, even) pairs of built-in IDT fingers.

**Supplementary note 2. Numerical simulation results of AO interactions**

To illustrate the reasonability of the designed TFLN-ChG hybrid waveguide platform in highly efficient AO modulation, we perform a numerical simulation of the refractive index change of the optical mode caused by the acoustic wave. Referring to the calculation method in Ref. 22 from the Loncar group, we estimate the refractive index changes induced by the photoelastic (PE) effect, moving boundary (MB) effect, and electro-optic (EO) effect. The detailed equations are same as that in Ref. 22. The calculation results for the fundamental optical modes and acoustic mode at 0.8317 GHz are displayed in Table S1 based on the current waveguide geometry (W_ChG_ = 1.6 μm, H_ChG_ = 850 nm, H_LN_ = 400 nm). Under this configuration, 83% mode energy is confined in the ChG film for TE00, and only approximately 15% energy is confined in the TFLN. It can be seen that the photoelastic effect of ChG film has the largest contribution (TE00 +83%, TM00 +93.5%) to the total refractive index change. For the TFLN, the electro-optic effect has small contribution (TE00 +17%, TM00 +9.35%). The photoelastic effect has slight contribution (TE00 +0.1%, TM00 −6.35%). Meanwhile, the calculation results illustrate that the fundamental TE mode has a larger refractive index change than the fundamental TM mode. This is why we choose the fundamental TE mode in our design. For the TFLN-ChG hybrid waveguide, the photoelastic property of the ChG has a larger contribution than that of the TFLN in the AO interaction. This is because the amorphous ChG film has a large photoelastic coefficient. The detailed photoelastic matrixes of the X-cut TFLN are provided. It can be concluded that the photoelastic effect of ChG film has dominant contribution to high modulation efficiency. In fact, the TFLN as slab component of the core of the hybrid rib waveguide is necessary with respect to the confinement of optical mode. In addition, the application of nonsuspended TFLN is beneficial to simplify the fabrication processes of heterogeneous-integrated AO modulator (e.g. no need for etching of LN, no need for deposition of oxide cladding, easy for waveguide end face coupling), and the excellent piezoelectric effect can still be maintained to efficiently excite SAWs.

Once the thickness of ChG waveguide is decreased, the electro-optic effect of TFLN would surpass the contribution of the photoelastic effect of ChG film. As an example, the numerical simulation results of AO interaction are shown in Table S2 based on a small waveguide geometry (W_ChG_ = 1.4 μm, H_ChG_ = 250 nm, H_LN_ = 400 nm). Under this configuration, 61% mode energy is confined in the TFLN for TE00, and approximately 31% energy is confined in the ChG film. On the contrary, it can be seen that the electro-optic effect of TFLN film has the largest contribution (TE00 +131.6%, TM00 −1920%) to the total refractive index change. The photoelastic effect of ChG film has small contribution (TE00 −17.3%, TM00 +494%). Therefore, the usage of the nonsuspended TFLN-ChG hybrid waveguide platform benefits us to flexibly adjust the device design so as to extend the functionalities of device.

Table S1. Numerical simulation results of AO interactions (W_ChG_ = 1.6 μm, H_ChG_ = 850 nm, H_LN_ = 400 nm)

| Optical  mode | Acoustic frequency (GHz) | *n*_MB_  ×10^−12^ | *n*_EO_  ×10^−12^ | *n*_PE_LN_  ×10^−12^ | *n*_PE_ChG_  ×10^−12^ | *n*_PE_total_  ×10^−12^ | *n*_total_  ×10^−12^ |
| --- | --- | --- | --- | --- | --- | --- | --- |
| TM00 | 0.8317 | −0.6426 | −1.6941 | 1.1502 | −16.9354 | −15.7851 | −18.1219 |
| TE00 | 0.8317 | −0.02279 | −4.0902 | −0.0228 | −19.8387 | −19.8615 | −23.9744 |

Table S2. Numerical simulation results of AO interactions (W_ChG_ = 1.4 μm, H_ChG_ = 250 nm, H_LN_ = 400 nm)

| Optical  mode | Acoustic frequency (GHz) | *n*_MB_  ×10^-12^ | *n*_EO_  ×10^-12^ | *n*_PE_LN_  ×10^-12^ | *n*_PE_ChG_  ×10^-12^ | *n*_PE_total_  ×10^-12^ | *n*_total_  ×10^-12^ |
| --- | --- | --- | --- | --- | --- | --- | --- |
| TM00 | 0.8320 | 2.4558 | −6.096 | 2.3885 | 1.5692 | 3.9577 | 0.3175 |
| TE00 | 0.8320 | 1.2595 | −14.5843 | 0.3272 | 1.9172 | 2.2443 | −11.0805 |

$$p_{LN}=\left(\begin{matrix} 0.076 & 0.138 & 0.138 & 0 & 0 & 0 \\ 0.149 & 0.045 & 0.096 & 0 & 0.055 & 0 \\ 0.149 & 0.096 & 0.045 & 0 & -0.055 & 0 \\ 0 & 0 & 0 & -0.067 & 0 & 0.055 \\ 0 & 0.12 & -0.12 & 0 & 0.019 & 0 \\ 0 & 0 & 0 & 0.12 & 0 & -0.025 \end{matrix}\right)$$

To reveal the dependence of AO interaction on geometries of hybrid waveguides, we conduct the numerical simulation for refractive index changes modulated by 0.8317GHz SAW at the different ChG waveguide sizes, which is shown in Fig. S2. With similar refractive indices between ChG and LN, we choose ChG waveguide width of 1.6 μm to match half acoustic wave wavelength and waveguide height of 850 nm to confine single optical mode, as denoted by the gray dot in Fig. S2. From the picture, it can be seen that the strength of AO interaction is related to the geometry of ChG waveguide. With the increase of waveguide width, the refractive index change of device decreases at fixed waveguide height of 850 nm. On the contrary, if the waveguide width is reduced to 1.4 μm, the AO interaction of device is expected to be further improved. Therefore, the careful engineering of waveguide geometry is beneficial to optimize AO modulation performance of device.


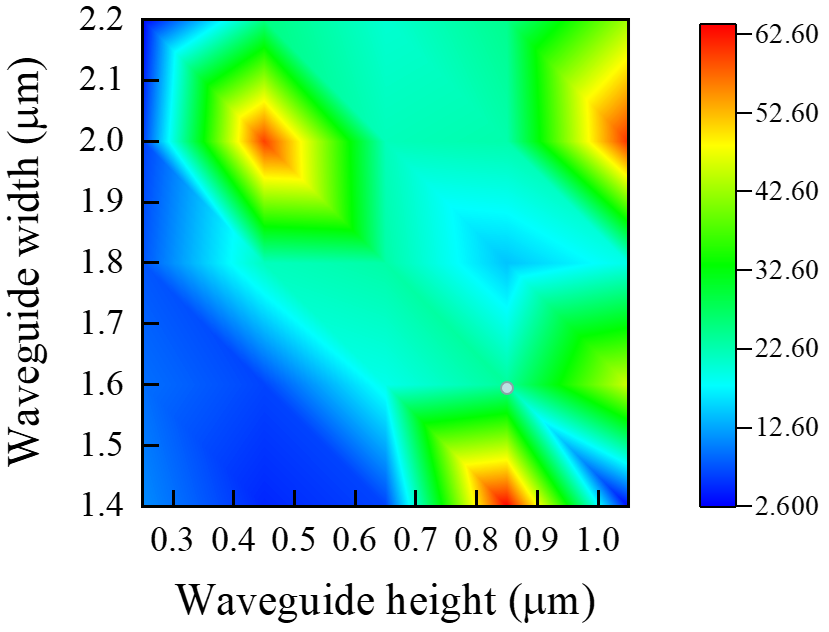


Fig. S2. Numerical simulation results of refractive index changes modulated by 0.8317 GHz SAW at different ChG waveguide geometries.

**Supplementary note 3. Derivation of the microwave-to-acoustic conversion efficiency**

To extract the microwave-to-acoustic conversion efficiency, we build a BVD model to analyze the effective acoustic wave energy participating in AO modulation, as shown in Fig. S3. The model includes four impedance parameters, i.e., R_s_, R_1_, C_e_ and R_a_. Herein, R_s_, R_1_ and C_e_ can be obtained by fitting the nonresonant normalized complex impedance of an IDT from 0.25 GHz to 1.25 GHz. The normalized complex impedance *z_L_* data can be calculated via the complex reflection coefficients from S_11_ measurements using the following formula:

$z_{L}=\frac{Z_{L}}{Z_{0}}=\frac{1+\Gamma}{1-\Gamma}$ (S1)

where *Z_L_* and Γ are the complex impedance and complex reflection coefficient of the IDT, respectively. *Z*_0_ is equal to 50 Ω. The load impedance Z_a_ can be derived via comparison with the total complex impedance of the effective circuit model and the measured complex impedance at the acoustic resonant frequency.

The microwave-to-acoustic conversion efficiency *η_IDT_* of the designed IDT is defined as follows:

$\eta_{IDT}\text{=}\eta_{EC}\times\left(1\text{-}\left|S_{11}\right|^{2}\right)$ (S2)

where *η_EC_* is the ratio of the power consumed on load Z_a_ to the power provided to the total circuit. 1-|S_11_|^2^ represents the proportion of microwave power applied to the total circuit removing the reflection of the input microwave power.


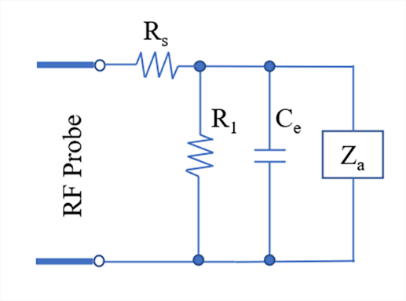


Fig. S3. Effective circuit model of the built-in IDT placed on the nonsuspended TFLN-ChG hybrid waveguide platform.

**Supplementary note 4. Comparison of the modulation efficiencies of the single arm and double arm simultaneously integrated AO modulators with the same MZI configuration (device A)**

To reveal the two times enhanced modulation efficiency in the double arm-modulated AO modulator compared to the counterpart with the single arm modulation configuration, we designed single arm and double arm simultaneously integrated AO modulators with the same MZI structure, as shown in Fig. 2(a). Figure S4(a) shows the optical transmission spectrum of the nonsuspended TFLN-ChG hybrid MZI. The ER of the device is estimated to be approximately 10 dB. Fixing the bias point at approximately 1548.6 nm, the DC optical power measurement is −14.5 dBm, and the opto-acoustic S_21_ measurements are −45.403 dB and −38.836 dB at 0.844 GHz for the single arm and double arm configurations, respectively, as presented in Fig. S4(b). Accordingly, the *V_π_* of the double arm (single arm) modulator is calculated to be 7.8 V (16.6 V) using equation 1 depicted in the manuscript. Given that the AO interaction length is 120 μm, the *V_π_L* of the double arm (single arm) modulator is calculated to be 0.094 V cm (0.2 V cm), demonstrating the twofold enhancement of the modulation efficiency.

In addition, we characterize the optical sidebands of the double arm-modulated AO modulator recorded at different input RF powers, as shown in Fig. S4(c). A third-order optical sideband appears when the RF power is increased to 13 mW. To trace the variations in the optical sidebands, we fit the data via the following formula:

 (S3)

 (S4)

 (S5)

where *P_opt-1_*, *P_opt-2_*, and *P_opt-3_* are the optical powers corresponding to the first-order, second-order, and third-order sidebands, respectively. *P_opt-in_* is the input optical power, and *IL* is the insertion loss of the MZI-based AO modulator. Δ*ϕ_m_* is the total phase shift caused by the AO interaction, and Δ*ϕ_i_* is the phase shift caused by the natural optical path difference Δ*L* of the MZI two-arm waveguides, which is described by the following:

 (S6)

 (S7)

where *L_m_* is the AO interaction length, which is equal to 120 μm in our modulator. Δ*n* is the effective refractive index change induced by the acoustic wave, and λ is the working wavelength of the device.


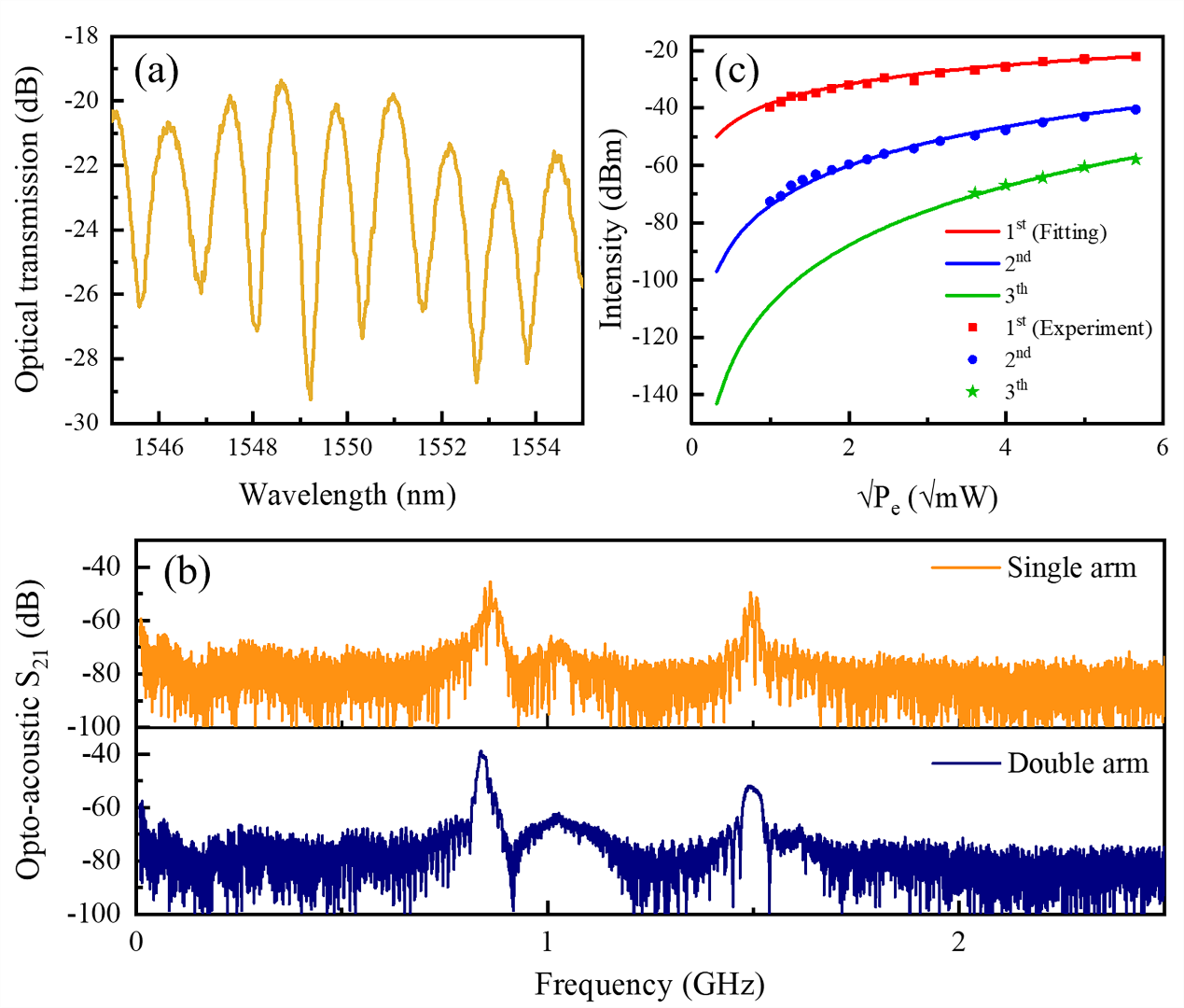


Fig. S4. **a** Optical transmission spectrum of the single arm and double arm simultaneously integrated AO modulators with the same MZI configuration. **b** Comparison of the S_21_ spectra of the AO modulators with the single arm and double arm modulation configurations. **c** Experimentally measured optical sidebands of the double arm-modulated AO modulator and the fitted results.

**Supplementary note 5. Estimation of photoelastic coefficients of Ge_25_Sb_10_S_65_ film**

Considering the different mechanical properties of Ge_25_Sb_10_S_65_ film in comparison with As_2_S_3_, the actual photoelastic coefficients (*p*_11_ ≈ *p*_12_) of amorphous Ge_25_Sb_10_S_65_ film in our experiments are calculated referring to the Ref. 33, which is presented in Fig. S5.

Fig. S5. Theoretical fitting of the experimental optical sideband powers and extraction of *α_p_* and *p*_11_ (*p*_12_).

Herein, the dominant photoelastic effect of ChG film is significantly concerned to approximately estimate the refractive index change induced by AO interaction, because 83% optical energy is confined in the ChG waveguide with thickness of 850 nm based on the TFLN-ChG hybrid platform. The quantitative refractive index change caused by the photoelastic effect of ChG film is given by

$\Delta n_{PE\_ChG}=-\frac{1}{2}n^{3}\frac{\iint(p_{11}S_{x}+p_{12}S_{z})\left|E(x,z)\right|^{2}dxdz}{\iint\left|E(x,z)\right|^{2}dxdz}$ (S8)

where *n* is optical mode index. *E* is defined as the electric field of optical mode. *S*_x_ and *S*_z_ are strain components at the interface between ChG and TFLN along crystal X and Z directions, respectively. Through the finite element simulation, *S*_x_ ≈ 0.44*S*_z_ is obtained to simplify the above equation.

According to the Ref. 33, the energy density of SAW in the ChG waveguide is approximately calculated by

$U=\frac{1}{2}(S_{x}C_{11}S_{x}+S_{x}C_{12}S_{z}+S_{z}C_{12}S_{x}+S_{z}C_{11}S_{z})$ (S9)

where *C*_11_ and *C*_12_ are elastic modulus of amorphous ChG film, which are dependent on the Young’s modulus G and Poisson’s ration ν of ChG film. By substituting *S*_z_ using 2.27*S*_x_, the energy of SAW in the ChG film is integrated by

$E_{ChG}=\iint Udxdydz=\frac{1}{4}CWLH\left|A\right|^{2}$ (S10)

where *C* is a constant. *W* and *H* are the width and height of ChG waveguide, respectively, and *L* is the aperture width of IDT. |*A*| is the amplitude of *S*_x_ stain field.

In the experiments, the generated effective energy of SAW can be approximately derived by

$E=\frac{(1-\left|S_{11}\right|^{2})P_{e}Q}{\omega_{a}}=\frac{E_{ChG}}{\zeta}$ (S11)

where *ω_a_* is angular frequency of acoustic wave, *P_e_* is the input microwave power. *Q* is natural quality factor of an IDT. *ζ* is energy ratio of SAW in the ChG waveguide relative to the total SAW energy. Using numerical simulation, this value is calculated to be 0.017, which means that only 1.7% acoustic wave energy is confined in the ChG waveguide to participate AO interaction. Accordingly, the |*A*| can be deduced by

$\left|A\right|=\sqrt{\frac{4\zeta Q(1-\left|S_{11}\right|^{2})P_{e}}{2\pi f_{0}CWLH}}$ (S12)

To relate the phase change in AO modulation with theoretical fitted *α_p_*, the following equation can be given by

 (S13)

where *φ*_AO_ is single-arm phase change of a MZI AO modulator, and *λ* is optical wavelength. Γ_AO_ is acousto-optic overlap factor, which is calculated to be 0.519. Compared with theoretical fitted *α_p_* = 0.18 rad/√mW, the *p*_11_ is estimated to be 0.238. The detailed parameters are displayed in Table S3.

Table S3. Parameters usage for *p*_11_ estimation of Ge_25_Sb_10_S_65_ film

| Parameter | Symbol | Value | Unit |
| --- | --- | --- | --- |
| Optical wavelength | $\lambda$ | 1550.455 | nm |
| Mode index | *n* | 2.1045 |  |
| Aperture length | *L* | 120 | μm |
| Acoustic wavelength | *Λ* | 3.2 | μm |
| Acoustic frequency | *f_0_* | 0.844 | GHz |
| AO overlap factor | Γ_AO_ | 0.519 |  |
| Waveguide width | *W* | 1.6 | μm |
| Height | *H* | 850 | nm |
| Acoustic Q factor | *Q* | 202.81 |  |
| Elastic modulus | *C*_11_ | 31.056 | GPa |
| Elastic modulus | *C*_12_ | 12.379 | GPa |
| Young’s modulus | *G* | 24 | GPa |
| Energy ratio | *ζ* | 0.017 |  |
| Poisson’s ratio | *ν* | 0.285 |  |

**Supplementary note 6. Evolution of the optical transmission spectrum of the double arm-modulated AO modulator with increasing RF power**

The distribution of the sideband spectrum closely depends on the choice of the bias point. If we change the bias point, the symmetry of the sideband spectrum would vary. Accordingly, if we sweep the bias point under different amounts of RF power, how does the optical transmission of the MZI change? With increasing input RF power, the optical transmission spectra of the double arm-modulated AO modulator are recorded to reveal the effect of the microwave power on light wave propagation, as shown in Fig. S6. The ERs of the transmission spectra of the device decrease with increasing RF power from 3 dBm to 15 dBm. This illustrates that the increased RF power induces abundant sideband modulation, leading to dissipation of light wave energy in the MZI. A lower RF power is thus beneficial for obtaining a sharp transmission spectrum.


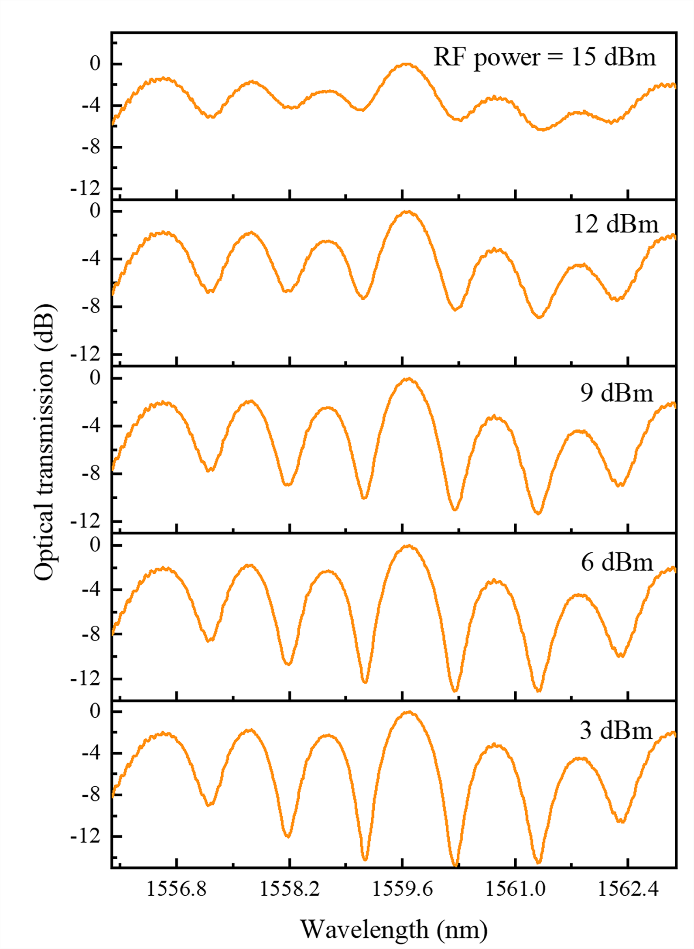


Fig. S6. Variation in the optical transmission spectrum of a built-in push-pull AO modulator with increasing input RF power.

**Supplementary note 7. Derivation of *α_p_* in the nonsuspended TFLN-ChG hybrid MZI-based AO modulator**

FOM *α_p_* represents the phase shift per unit square root acoustic power, which can be calculated by the following:

 (S14)

where |Δ*ϕ_m_*| is the total phase shift caused by the acoustic wave and *P_a_* is the microwave power minus the IDT reflection. When a π phase shift occurs, the corresponding acoustic power is denoted as *P_a-π_*, which is given by the following:

$P_{a-\pi}=P_{e-\pi}\times\left(1\text{-}\left|S_{11}\right|^{2}\right)$ (S15)

where *P_e-π_* is the input RF power corresponding to the π phase shift, which can be determined by the following:

$P_{e-\pi}=\frac{V_{\pi}^{2}}{2\times50}$ (S16)

**Supplementary note 8. Illustration of the fabrication processes of the built-in push-pull AO modulator in the nonsuspended TFLN-ChG hybrid waveguide platform**


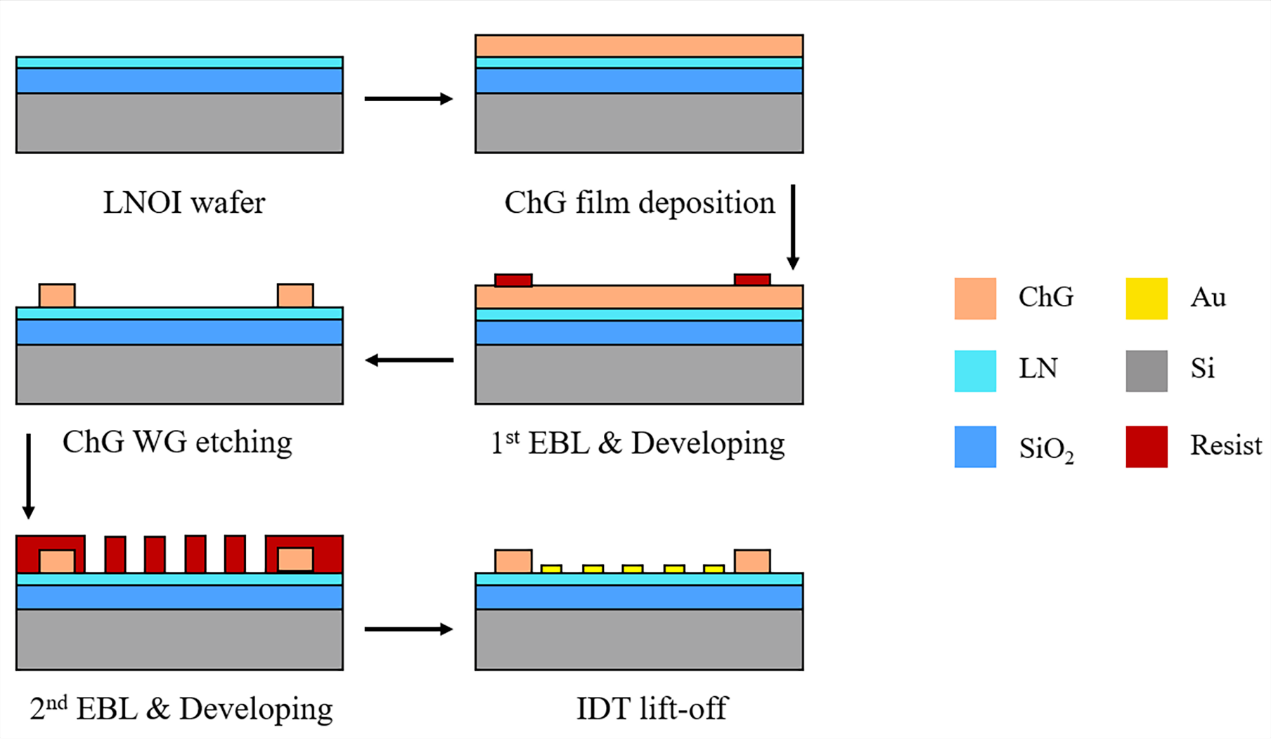


Fig. S7. Schematic of the fabrication processes of the built-in push-pull AO modulator based on the nonsuspended TFLN-ChG hybrid waveguide platform.
